# Clinical Characteristics of Colorectal Cancer Patients in terms of Selected Platelet Indices

**DOI:** 10.1155/2020/6145604

**Published:** 2020-10-08

**Authors:** Angelika Copija, Ewa Nowakowska-Zajdel, Karolina Janion, Katarzyna Walkiewicz

**Affiliations:** ^1^Department of Nutrition-Related Disease Prevention, Department of Metabolic Disease Prevention, School of Public Health in Bytom, Medical University of Silesia, Katowice, Poland; ^2^Department of Clinical Oncology, Regional Specialised Hospital No 4, Bytom, Poland

## Abstract

Mounting evidence suggests that inflammation, immune response, and coagulation status determine many processes during the carcinogenesis pathway in colorectal cancer (CRC). Inflammation strongly promotes tumor formation, progression, and metastasis. The systemic inflammatory response (SIR) may be reflected by simple indicators evaluated on the basis of peripheral blood morphology parameters. The indices are easily obtained by the peripheral blood test and could be promising biomarkers for CRC. We present the results of the retrospective study evaluating the potential relation between the platelet indices (platelet count (PC), platelet-to-lymphocyte ratio (PLR), neutrophil platelet score (NPS), mean platelet volume (MPV), and MPV/PC ratio) and the clinicopathological features of CRC patients. The study included 247 patients (104 males and 143 females) aged 39-87 years with CRC stages II-IV. The complete blood counts with the automated differential counts were performed prior to the qualification to systemic treatment. High PC, high PLR, and NPS 0 were associated with older age and higher BMI of the patients. No link between the analyzed platelet indices and histological grade of the tumor, primary tumor location, and gender was noted. The patients aged ≥65 years were characterized by the higher MPV/PC ratio than the younger population. We observed a trend to the higher MPV/PC ratio among the patients with excessive body weight defined by BMI compared to BMI within normal limits. A higher frequency of PC > 400, NPS 1 and 2, and a trend to more frequent PLR ≥ 150 were observed in the subgroup with metastatic disease compared to individuals with CRC stages II and III. The presented results expand the knowledge on potential association between SIR parameters and other clinicopathological factors that should be considered during interpreting the prognostic and predictive value of the inflammation parameters.

## 1. Introduction

In 1863, Rudolf Virchow hypothesized that chronic inflammation could sustain cell proliferation contributing to cancerogenesis [1]. Mounting evidence established the role of inflammatory and thrombotic processes in all stages of carcinogenesis in colorectal cancer. The local cancer-related inflammation is reflected in the systemic inflammatory response (SIR).

Besides their physiological role in hemostasis, platelets contribute various pathological processes including inflammation, atherosclerosis, and cancer metastasis via the release of cytokines and chemokines and expression of several adhesion receptors [2]. Tumor cells secrete a plethora of bioactive mediators contributing to platelet activation, e.g., cysteine proteinases, ADP [3] or IL-6 [4]. Platelets may be activated by the production of thrombin stimulated by the tissue factor, upregulated in the oxidative stress in the tumor microenvironment [5]. The colorectal cancer cells and activated platelets may interact using *α*IIb*β*3 structures or via P-selectin [5]. Activated platelets secrete several tumor growth and proangiogenic factors involved in the cancerogenesis, including transforming growth factor *β*, platelet-derived growth factor, vascular endothelial growth factor, epidermal growth factor, and angiopoietin [6]. Circulating tumor cells avoid the immune surveillance and promote their extravasation and metastasis formation by being coated by blood platelets making them unrecognizable for the natural killer cells [5, 7].

The potential prognostic role of the combinations of the SIR parameters (such as lymphocytes, neutrophils, and platelets obtained by the peripheral blood test), including the platelet-to-lymphocyte ratio (PLR), neutrophil platelet score (NPS), or mean platelet volume (MPV) to platelet count (PC) ratio, was investigated in various cancers, including CRC [8–10]. However, majority of the studies focused on the primary operable cancer [11–15]; some of them observed patients with advanced diseases [16–18] or patients in all clinical stages [19]. The relation between platelet indices and clinical features is still inconsistent. Moreover, the interpretation is difficult because comorbidities affecting this imbalance may be important in the pathophysiology of SIR.

The aim of the study is to explore the potential relation between the platelet indices (PC, PLR, NPS, MPV, and MPV/PC) and the clinicopathological features of colorectal cancer patients.

## 2. Material and Methods

We conducted the retrospective observational study. Medical records of 247 consecutive patients with histologically confirmed colorectal cancer were evaluated. The study group included patients with disease stages II-IV prior to qualification to systemic treatment (adjuvant or first-line palliative treatment). The established exclusion criteria included the following: other active or past cancers except squamous cell carcinoma of the skin, underwent chemotherapy for CRC during the past 6 months, diagnosed infectious disease (hepatitis B and C, HIV infection or AIDS, and tuberculosis), systemic diseases requiring long-term immunosuppression, cancer cachexia, and no patient consent to participate in the study.

Patients' demographics and clinical and pathological characteristics were collected on the basis of medical history. The peripheral venous blood samples were collected from each patient from a single puncture at a routine blood test necessary for qualification for chemotherapy. Whole blood samples were collected in EDTA-containing tubes, and complete blood counts with automated differential counts were performed.

PLR was calculated by dividing PC by the absolute neutrophil count (NC). Similarly, the MPV/PC ratio was calculated by dividing MPV by the platelet count. NPS was calculated as follows: patients with a neutrophil count ≤ 7.5 × 10^9^/L and platelets ≤ 400 × 10^9^/L scored 0, patients with neutrophils > 7.5 × 10^9^/L or platelets > 400 × 10^9^/L scored 1, and patients with both neutrophils > 7.5 × 10^9^/L and platelets > 400 × 10^9^/L scored 2 [20].

### 2.1. Statistical Analysis

Data were presented as mean ± standard deviation or median (interquartile range) for continuous variables and frequency or rate for categorical variables. The normal distribution in the analyzed population was tested with the Shapiro-Wilk test. The Mann*-*Whitney *U* test and the Kruskal-Wallis *H* test were used to determine the significance of between-group differences. All statistical analyses were carried out using the Statistica version 13 software package (StatSoft).

## 3. Results

The study group contains 247 patients aged 39-87 years treated in the Department of Clinical Oncology in the period from January 2016 to July 2019. Baseline characteristics of the study group are presented in Table 1.

We observed no association between the analyzed platelet indices and the histological grade of the tumor, primary tumor location (Table 2), and gender.

Patients with metastatic disease had higher WBC and NC compared to individuals with CRC stages II and III after radical resection (Table 3). We noted a higher frequency of PC > 400, NPS 1 and 2, and a trend to more frequent PLR ≥ 150 in the subgroup with stage IV disease (Table 4).

High PC (defined according to the literature as PC > 400 [9, 14, 21, 22]) and high PLR (defined as PLR > 150 [8, 9, 13–15, 18, 22]) were linked with younger age and lower BMI of the patients (Table 4). Similarly, NPS 0 was related with older age (*p* = 0.0010) and higher BMI (*p* = 0.0186) of patients compared to the subgroup with NPS 1 and 2. No link between MPV and patients' BMI or age was observed. There was a trend to the lower MPV/PC ratio among patients with normal weight compared to overweight and obese patients (0.028 (0.021–0.039) vs. 0.030 (0.023–0.040) vs. 0.033 (0.025–0.046) accordingly, *p* = 0.052). The MPV/PC ratio was higher in patients aged ≥65 years than in the younger population (0.032 (0.024–0.044) vs. 0.028 (0.021–0.037) accordingly, *p* = 0.009).

## 4. Discussion

Platelet involvement is observed in almost every step of the metastatic process [5]; hence, platelet indices are potential interesting markers in the metastatic disease. Platelets induce circulating tumor cell epithelial-mesenchymal migration and facilitate tumor cell extravasation and metastasis formation [6, 23, 24]. Experimental studies on animal models indicated the inhibition of pulmonary metastasis by induced thrombocytopenia, which can be reconstituted by platelet infusion [25].

Contrarily, lymphocytes mediate tumor growth control and were inversely related with tumor proliferation and invasiveness [26]. Differentiated CD8+ T cells induce cytotoxic T cell killing and apoptosis. CD4+ T cells play a crucial role in the antitumor immune response. A reduced lymphocyte level facilitates tumor metastatic potential [27]. A decrease in tumor-infiltrating lymphocytes is postulated to be associated with poor survival in CRC [28]. Although no direct research has demonstrated that the peripheral lymphocyte count correlates with the number of tumor-infiltrating lymphocytes, some studies have postulated an association between them [9].

An elevated PLR is a result of an increased number of platelets and/or a decreased number of lymphocytes. Results of a meta-analysis conducted by Chen et al. suggest the association between increased PLR and inferior clinical features of CRC such as poorly differentiated tumor (OR = 1.51; 95% CI, 1.26-1.81; *p* < 0.001), higher tumor stage (OR = 1.25; 95% CI, 1.05-1.49; *p* = 0.012), lymphovascular invasion (OR = 1.25; 95% CI, 1.09-1.43; *p* = 0.001), and recurrence of CRC (OR = 2.78; 95% CI, 1.36-5.68; *p* = 0.005) [8], suggesting that PLR could be feasible for tumor staging. Another meta-analysis by Huang et al. confirms the relation between elevated PLR and more advanced clinical stage, pT category, and degree of differentiation; however the association between lymph node metastasis, lymphatic and venous invasion, and PLR was not observed [9]. Our results do not support these findings; however, in most of the studies included in the meta-analyses, PLR was obtained before the surgery in patients with early-stage CRC while our results are obtained in the population with stage II-IV disease and the majority of the patients had underwent surgery before.

Previous studies showed the association between high PLR and female gender [8] and cancer location in the colon vs. rectum [8, 29], which was not confirmed in our results.

The relation between platelet indices and CRC patients' nutritional status has not been sufficiently examined. Obesity is the well-established risk factor for colorectal cancer. One-third of the patients in the analyzed population were overweight, and one-fifth were obese. We observed lower PC and a trend to lower PLR in patients with excess BMI. These results are puzzling considering the previously described link between increased PC and metabolic syndrome in adults [29]. Increased adipose tissue interferes with platelet function directly by producing adipokines, such as leptin and adiponectin, and inducting chronic systemic low-grade inflammation [30]. Adipose tissue in obese individuals is infiltrated with increased numbers of macrophages producing proinflammatory cytokines [31]. High concentrations of adiposity-related inflammatory factors, i.e., tumor necrosis factor-alpha (TNF-*α*), interferon-*γ*, and interleukin- (IL-) 1, 6, 8, and 10, have been detected both in the adipose tissue and blood [32]. Inflammatory markers such as the high-sensitivity C-reactive protein (hsCRP) and white blood cell (WBC) count have been linked with the degree of obesity expressed as the BMI [33], waist circumference (WC) [34], and waist-hip ratio [35]. Some authors suggest the association between the visceral adipose tissue area and the WBC, hsCRP, and NLR levels (but not PLR), whereas no similar observation was confirmed for subcutaneous adipose tissue areas [36]. Recently published findings indicate significantly higher PLR in the colorectal cancer patients with the metabolic syndrome; however, the results were obtained in the group of patients before surgical treatment [29]. On the other hand, results of the population-based survey conducted in almost 40,000 US citizens aged ≥60 years showed that elevated PLR values were significantly associated with the sarcopenia status and negatively associated with the skeletal muscle index [37]. Considering the above results and the fact that the majority of patients with CRC are aged ≥60 years and are often affected by excessive body weight, the interpretation of the described links and the potential diagnostic significance of platelet indices require further research. It remains unclear if the inflammatory processes associated with exceeding from normal weight to overweight in cancer-free individuals differ in terms of pathophysiology from the inflammatory processes associated with colorectal cancer development.

MPV reflects platelet size and is a surrogate of platelet activation [12, 38]. Decreased MPV may be the effect of increased large platelets in terms of inflammation, possibly because larger platelets are more responsive to stimulation and plenty of larger platelets are selectively degraded in the environment of the neoplasm [10]. Additionally, the inverse relationship between MPV and PC suggests that these two variables should be interpreted as a ratio rather than being used alone [10]. Recent studies showed the higher MPV levels in colorectal cancer patients compared to the control group [12, 19] and the reduction of MPV after surgical tumor resection [12]. Based on these observations, MPV could be tested as a potential marker of surveillance in postoperative CRC patients. However, the data on the possible prognostic role of MPV is ambiguous. Some evidence suggests the association between increased MPV and poor overall survival in patients undergoing surgical treatment [39]. On the other hand, decreased pretreatment MPV was showed to predict worse prognosis in the population with advanced CRC qualified to first-line palliative chemotherapy [16]. In the present study, we observed no differences in MPV in CRC patients through TNM stages I-IV which is in agreement with previously published data [19]. Wu et al. described the significant difference in MPV/PC and TNM stage of CRC (I/II vs. III/IV) and in terms of lymph node invasion [10], which was not confirmed in our results. We noted no link between the MPV or MPV/PC ratio and the other inferior clinical features of CRC.

We analyzed NPS postulated by Watt et al. as a promising marker predicting survival in patients undergoing potentially curative surgery for CRC, independent of the TNM stage [20]. In the analyzed population, NPS 1 and 2 were more frequent in patients with metastatic disease. However, due to restrictive criteria in the calculation of NPS, NPS2 was found in a small percentage of cases; therefore, the results of the subgroup analysis within a small study group do not achieve adequate statistical power. Although single reports regarding the use of this parameter in the diagnosis of other gastrointestinal neoplasms have been published (in patients with operable gastric cancer [40] and metastatic pancreatic cancer [41]), no prognostic value of NPS was proven.

## 5. Conclusions

Patients with metastatic disease were more frequently characterized by high PC, high PLR, and NPS 1 and 2 compared to stage II-III disease. The relation between PC, PLR, and NPS and patients' age and BMI was observed. We noted no significant link between analyzed clinicopathological parameters in terms of MPV and MPV/PC.

The interpretation of the present study has a number of possible limitations. First, this was a single-center retrospective study and additional larger validation studies are needed to confirm our results. Second, the data on overall survival is not available yet. Further prospective studies are warranted to assess the exact role of platelet indices in colorectal cancer. Comorbidities were not analyzed because in the authors' opinion the interpretation would be difficult. Not only the presence of comorbidities but also their duration, treatment, and patient lifestyle should be considered. The authors focused on the analysis of SIR with respect to age, sex, BMI, stage, and location of the primary tumor, and most patients in the study group were over 50 years old.

The platelet indices including PLR, NPS, MPV, and MPV/PC may be a promising diagnostic biomarker for CRC. They are inexpensive and widely available. However, many possible medical conditions, including obesity or inflammatory diseases, can potentially affect platelets. Hence, it is particularly important to better understand potential association between SIR parameters and other clinicopathological factors, which should be taken into account when interpreting the results of studies on the prognostic and predictive value of inflammation parameters.

## Figures and Tables

**Table 1:** General characteristics of the study group, *n* = 247.

Variable	*n* (%)
Age (years)	
≤65	105 (42.5)
>65	142 (57.6)
Gender	
Male	104 (42.1)
Female	143 (57.9)
BMI (kg/m^2^)	
20-25	109 (44.1)
25-30	83 (33.6)
≥30	55 (22.3)
Grade (*n* = 200)	
I	16 (8)
II	138 (69)
III	46 (23)
Stage	
II	51 (20.7)
III	65 (26.3)
IV	131 (53.0)
Primary tumor location	
Right colon	72 (29.1)
Left colon	121 (49.0)
Rectum	54 (21.9)

**Table 2:** Characteristics of the study group according to primary tumor location.

Variable	Total	Primary tumor location			*p*
		RCC	LCC	RC	
Age (years)	66.02 ± 9.20	68.67 ± 7.24	65.13 ± 9.72	64.48 ± 9.74	0.0319
BMI (kg/m^2^)	26.24 ± 4.65	26.72 ± 4.82	25.74 ± 4.71	26.47 ± 4.21	0.218
WBC (×10^9^/L)	6.90 (5.65–8.81)	6.56 (5.56–8.96)	7.55 (5.80–9.12)	6.60 (4.40–8.56)	0.094
NC (×10^9^/L)	4.38 (3.27–6.68)	3.94 (2.99–5.38)	4.54 (3.34–6.03)	4.34 (3.52–5.11)	0.203
PC (×10^9^/L)	276 (221–342)	276 (216–342)	276 (229–341)	277 (216–351)	0.678
PLR	158.5 (119.4–237.1)	153.8 (11.6–209.1)	155.4 (121.8–219.6)	174.1 (122.8–308.5)	0.156
MPV (fL)	8.1 (7.1–10.3)	8.2 (7.1–10.6)	8.4 (7.2–10.5)	7.9 (7.0–9.8)	0.351
MPV/PC ratio	0.030 (0.023–0.040)	0.030 (0.023–0.045)	0.031 (0.023–0.041)	0.030 (0.021–0.038)	0.629

**Table 3:** Characteristics of the study group according to clinical stage of CRC.

Variable	Total	Clinical stage			*p*
		II (*n* = 51)	III (*n* = 65)	IV (*n* = 131)	
Age (years)	66.02 ± 9.20	65.29 ± 9.11	65.71 ± 9.66	66.46 ± 9.05	0.921
BMI (kg/m^2^)	26.24 ± 4.65	26.04 ± 4.44	26.78 ± 4.92	26.05 ± 4.61	0.695
WBC (×10^9^/L)	6.89 (5.65–8.81)	6.13 (5.47–8.01)	6.28 (5.03–7.77)	7.94 (6.10–10.08)	<0.0001
NC (×10^9^/L)	4.38 (3.27–5.68)	3.72 (2.69–4.70)	3.56 (2.89–4.77)	4.80 (3.74–6.48)	<0.0001
PC (×10^9^/L)	276 (221–342)	273 (229–342)	264 (219–323)	288 (220–374)	0.152
PLR	158.5 (119.4–237.1)	138.2 (106.4–207.5)	150.8 (126.0–237.0)	165.1 (125.0–250.9)	0.158
MPV (fL)	8.1 (7.1–10.3)	8.9 (7.1–10.2)	7.9 (7.2–9.9)	8.3 (7.1–10.5)	0.742
MPV/PC ratio	0.030 (0.023–0.040)	0.029 (0.025–0.043)	0.032 (0.024–0.041)	0.031 (0.021–0.039)	0.308

**Table 4:** Characteristics of subgroups depending on PC, PLR, and NPS.

Variable	PC		*p*	PLR		*p*	NPS			*p*
	<400	>400		<150	≥150		0	1	2	
	*n* (%)	*n* (%)		*n* (%)	*n* (%)		*n* (%)	*n* (%)	*n* (%)	
Age			0.0028			0.0016				0.0004
<65	81 (77.1)	24 (22.8)		35 (33.3)	70 (66.7)		73 (69.5)	23 (21.9)	9 (8.6)	
>65	129 (90.8)	13 (9.1)		76 (53.5)	66 (46.5)		127 (89.4)	10 (7.1)	5 (3.5)	
Gender			0.336			0.377				0.090
Male	92 (87.6)	13 (12.4)		44 (41.9)	60 (58.1)		88 (83.8)	15 (14.3)	2 (1.9)	
Female	118 (83.1)	24 (16.9)		68 (47.6)	75 (52.4)		112 (78.9)	18 (12.7)	12 (8.4)	
BMI			0.0278			0.0796				0.194
≤25	87 (79.1)	23 (20.9)		42 (38.2)	68 (61.8)		85 (77.3)	15 (13.6)	10 (9.1)	
25-30	71 (86.8)	11 (13.4)		36 (47.0)	44 (53.0)		66 (80.5)	13 (15.9)	3 (3.6)	
≥30	52 (94.6)	3 (5.4)		31 (56.4)	24 (43.6)		49 (89.1)	5 (9.1)	1 (1.8)	
Tumor location			0.172			0.577				0.172
RCC	66 (91.7)	6 (8.3)		34 (47.2)	38 (52.8)		63 (87.5)	8 (11.1)	1 (1.4)	
LCC	99 (81.8)	22 (18.2)		57 (47.1)	64 (52.9)		94 (77.7)	16 (13.2)	11 (9.1)	
RC	45 (83.3)	9 (16.7)		21 (38.9)	33 (61.1)		43 (79.6)	9 (16.7)	2 (3.7)	
Grade			0.324			0.596				0.760
I	15 (93.8)	1 (6.2)		6 (37.5)	10 (62.5)		14 (87.5)	2 (12.5)	0 (0)	
II	119 (86.2)	19 (13.8)		70 (50.7)	68 (49.3)		115 (83.3)	16 (11.6)	7 (5.1)	
III	43 (93.5)	3 (6.5)		22 (47.8)	24 (52.2)		41 (89.1)	4 (8.7)	1 (2.2)	
Clinical stage			0.0122			0.063				0.0046
II	47 (92.2)	4 (7.8)		29 (56.9)	22 (43.1)		47 (92.2)	4 (7.8)	0	
III	60 (92.3)	5 (7.7)		32 (49.2)	33 (50.8)		58 (89.3)	6 (9.2)	1 (1.5)	
IV	103 (78.6)	28 (21.4)		51 (38.60)	81 (61.4)		95 (72.5)	23 (17.6)	13 (9.9)	

## Data Availability

Requests for additional data or for support with reusing the data should be emailed to the authors, who can be contacted at angelika.copija@gmail.com.
